# To Be Safe

**Published:** 1877-01

**Authors:** 


### TO BE SAFE,

BE in proper places at proper times, and mind your own business. With such restrictions, we believe human life is as safe in New York as in any other city on the globe. Nine times out of ten, the reports of persons who have fared badly, or who have fallen into the hands of the Philistines, indicate one of two things—an out-of-the-way place, or an unseasonable hour. A young man attends a private party, leaves at two o'clock in the morning, and is heard of no more. Another visits New York, and, with his pockets full of money, promenades the river streets alone, an hour or two after dark. It is at one o'clock in the morning that a man was "*knocked down, and robbed of several thousand dollars*," with no trace of the robbers—unless you get him to tell where and what he was doing between the hours of decent bed-time and those of early morning. The fact is, a good many of the robberies fathered on large cities never took place. The assignation and the gaming table are the maelstroms of half the "lost pocket-books" of the city morning newspapers.

